# Short-chain fatty acids in the tumor microenvironment: from molecular mechanisms to cancer therapy

**DOI:** 10.7150/thno.119304

**Published:** 2026-01-01

**Authors:** Yan Xiang, Ao Du, Zhen Wang, Hongyuan Pan, Kefei Yuan

**Affiliations:** Department of Liver Surgery, State Key Laboratory of Biotherapy and Cancer Center, West China Hospital, Sichuan University and Collaborative Innovation Center of Biotherapy, Chengdu, China.

**Keywords:** short-chain fatty acids, tumor microenvironment, cancer therapy, molecular mechanisms, immune modulation

## Abstract

Short-chain fatty acids (SCFAs), including acetate, propionate, and butyrate, serve as pivotal metabolites within the tumor microenvironment (TME), playing essential roles in modulating tumor progression. Although the biological functions and mechanisms of SCFAs in the TME show some overlap, each SCFA also exerts some distinct regulatory effects on tumors and TME. Notably, even a single SCFA may exhibit pleiotropic effects across different cancer types or under varying conditions within the same malignancy. Consequently, according to the different metabolic microenvironment of patients, precise modulation of SCFA levels could effectively suppress tumor progression. Furthermore, SCFAs have been shown to potentiate the therapeutic efficacy of immunotherapy, radiotherapy, and chemotherapy. This review systematically outlines the sources, biological functions, and mechanisms of different SCFAs in the TME, while exploring potential therapeutic strategies based on SCFA modulation. These insights offer novel perspectives and directions for future research and clinical cancer therapy.

## 1. Introduction

Cancer is one of the leading causes of global mortality 1. In recent years, with a deeper understanding of cancer pathogenesis, a growing number of therapeutic options have become available. However, most current cancer therapies primarily target tumor cells, often overlooking the role of the TME in cancer treatment. The TME is composed of various cellular and non-cellular components within the tumor niche 2. In the past few decades, the importance of the TME in dynamically regulating cancer progression and influencing treatment outcomes has been widely recognized 3-5. Paget compared the relationship between tumor cells and the TME to that of seeds and soil, emphasizing that tumor cell growth depends on the support of the TME 6,7. As the “soil” for tumor growth, non-cellular components within the TME, such as chemokines cytokines, and growth factors, play crucial roles in regulating cancer progression 8. Furthermore, nutrients, as essential non-cellular components for maintaining cellular functions within the TME, also perform highly complex roles in this microenvironment 9.

Glucose, amino acids, and lipids are currently regarded as the principal nutrients within the TME 10,11. Unlike glucose and amino acids, lipid research has lagged due to their structural diversity and unique physicochemical properties 12,13. However, advances in lipidomics have deepened our understanding of lipid functionality 14-16. As the basic skeleton of lipids, fatty acids (FAs) exert diverse biological effects in the TME 17,18. Compelling evidence indicates that FA synthesis, uptake, and metabolism profoundly influence cancer progression, and FAs also serve as signaling molecules to activate pathways governing cellular activities 13. These findings highlight the necessity to explore the role of FA in tumor progression.

FAs are categorized by carbon chain length into short-chain fatty acids (SCFAs; <6 carbons), medium-chain fatty acids (MCFAs), and long-chain fatty acids (LCFAs) 19. SCFAs, including acetate, propionate, and butyrate, are primarily generated through gut microbial fermentation of dietary fibers (e.g., inulin, pectin, resistant starch). Then they cross the intestinal epithelial cells, enter the bloodstream, and are transported to the TME via the circulatory system 20-22. Their roles in the TME have attracted significant attention. Some studies have shown that SCFAs can directly either inhibit or promote tumor cell growth depending on the context 23,24. Additionally, other research has indicated that SCFAs can modulate the function and fate of immune cells within the TME, thereby influencing tumor progression 25,26.

This review systematically delineates the origins, biological functions, and molecular mechanisms of SCFAs in the TME. We also explore SCFA-based therapeutic strategies, aiming to provide novel insights and translational directions for cancer treatment.

## 2. Acetate

### 2.1 Sources of Acetate

**Figure 1:**
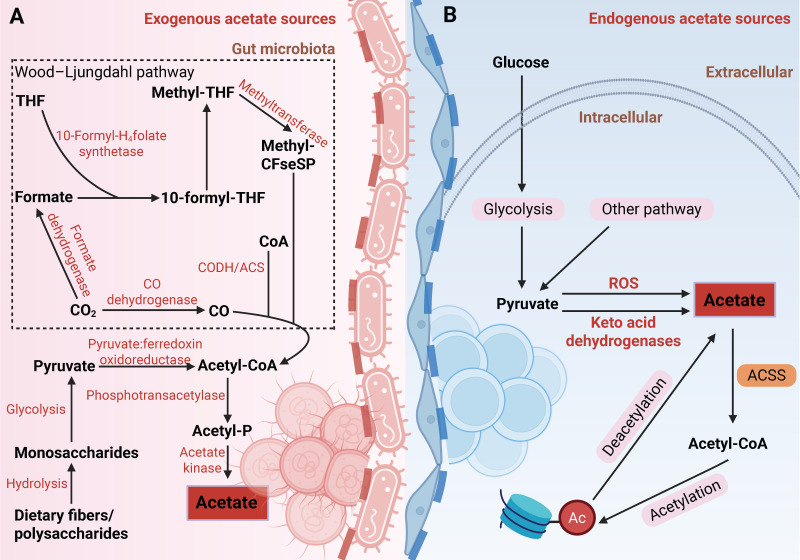
** Schematic diagram of acetate production by gut microbiota and mammalian cells in the human body.** (A) Exogenous acetate sources: Indigestible dietary fibers or polysaccharides are hydrolyzed into monosaccharides, which then enter the gut microbiota. These monosaccharides undergo glycolysis to produce pyruvate, which is subsequently converted to acetyl-CoA by pyruvate:ferredoxin oxidoreductase. Acetate is then synthesized via phosphotransacetylase and acetate kinase. Alternatively, the Wood-Ljungdahl pathway reduces CO₂ to acetate through a series of enzymatic reactions involving CODH/ACS, formate dehydrogenase, and 10-formyl-H_4_folate synthetase, ultimately leading to the formation of acetyl-CoA and acetate. (B) Endogenous acetate sources: Glucose-derived pyruvate undergoes decarboxylation, either through ROS-mediated or keto acid dehydrogenase-driven pathways, to produce acetate, which is subsequently converted to acetyl-CoA by ACSS for histone acetylation. Deacetylation of histones releases acetate, thereby completing the metabolic cycle. Abbreviations: ACSS, acetyl-CoA synthetase; CODH, carbon monoxide dehydrogenase; ROS, reactive oxygen species; THF, tetrahydrofolate.

Acetate in the human body originates from exogenous uptake and endogenous synthesis (Figure 1). Exogenous acetate is primarily generated through the fermentation of dietary fibers by gut microbiota. Acetogenic bacteria are strict anaerobes. These bacteria generate acetate via two major pathways: (1) carbohydrate metabolism, where glucose is converted to pyruvate, which is then converted into acetyl-CoA and further transformed into acetate 27,28; (2) the Wood-Ljungdahl pathway, where certain bacteria reduce CO₂ to generate acetate 29,30.

As research progresses, our understanding of the sources of acetate in both physiological and pathological states has become more comprehensive. Moving beyond exogenous uptake, the mechanisms of endogenous acetate production have also emerged as a growing research focus. In humans, endogenous acetate can be generated through pathways such as glucose metabolism and protein deacetylation. Liu et al. discovered that pyruvate, a product of glucose metabolism, can be decarboxylated to acetate through reactive oxygen species (ROS) or a novel enzymatic activity of alpha-keto acid dehydrogenase 31. Additionally, chromatin serves as an acetate reservoir, releasing acetate through histone deacetylation 32,33. These processes help alleviate metabolic stress in the TME, such as nutrient deprivation and acidosis, and are of significant pathological relevance. For instance, under nutrient-restricted conditions, glioma cells secrete acetate derived from glucose metabolism, promoting the proliferation of astrocytes 34. In an acidic environment, cancer cells exhibit global deacetylation, and the released acetate anions are co-transported with protons via monocarboxylate transporters (MCTs), preventing further acidification of the intracellular pH 35. Collectively, acetate in the TME originates from diverse metabolic pathways, highlighting its critical role as a key metabolite in the metabolic reprogramming of tumors.

### 2.2 Acetate and Cancer

Studies have shown that acetate can directly affect various tumors, including colorectal cancer (CRC), pancreatic ductal adenocarcinoma (PDAC), and hepatocellular carcinoma (HCC) 32,36,37. The regulatory effects of acetate on tumors exhibit “double-edged sword” properties. For example, in non-alcoholic fatty liver disease-related hepatocellular carcinoma (NAFLD-HCC), acetate can inhibit tumor progression by activating the G protein-coupled receptor 43 (GPR43) 38; whereas in glioblastoma, acetate supports tumor growth by sustaining the metabolic demands of cell proliferation 39. Mechanistically, the antitumor effects of acetate are primarily mediated through two pathways: receptor-mediated signaling inhibition and metabolic interference-induced cell damage. Upon binding to GPR43, acetate inhibits the IL-6/JAK1/STAT3 signaling pathway, thereby blocking NAFLD-HCC initiation and growth 38. Additionally, acetate can directly induce oxidative stress and mitochondrial dysfunction in CRC cells, activating caspase-dependent apoptosis pathways 40,41.

**Figure 2:**
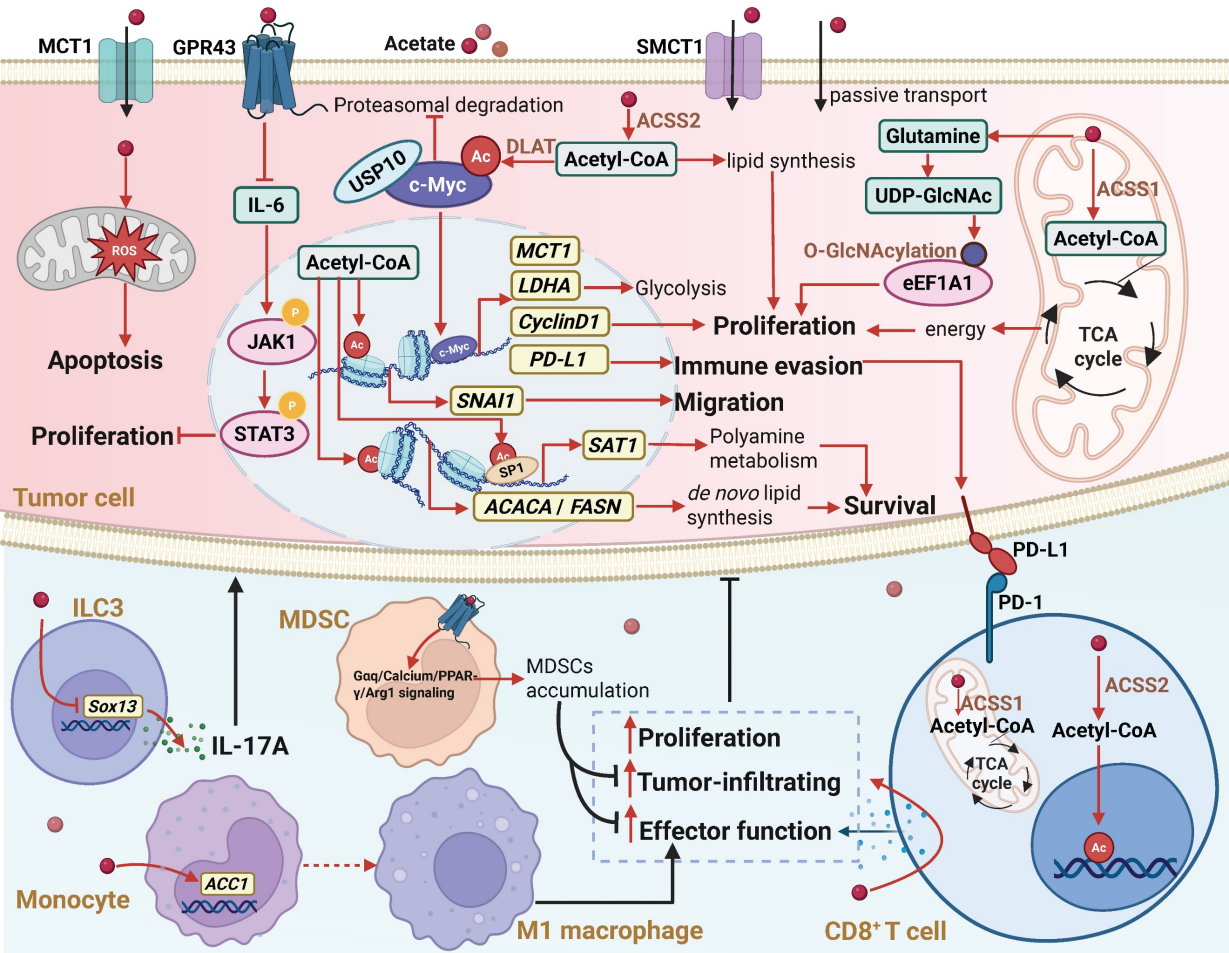
** The biological functions and mechanisms of acetate in the TME.** In the TME, acetate can regulate various molecular mechanisms by either binding to the GPR43 receptor or entering cells through monocarboxylate transporters MCT1 and SMCT1, or passive transport. Extracellular acetate binds to GPR43, activating the receptor and triggering a GPR43-mediated cellular signaling cascade. Acetate that enters the cell can replenish the intracellular acetyl-CoA pool, leading to increased histone acetylation and enhanced gene transcription. Additionally, acetate can serve as an alternative energy source for the tricarboxylic acid cycle or act as a key precursor for the biosynthesis of macromolecules such as lipids. Through these mechanisms, acetate regulates cancer cell proliferation, metastasis, and immunogenicity, while also affecting the proliferation, antitumor function, and tumor infiltration of immune cells in the TME. Abbreviations: ACSS1, acetyl-CoA synthetase 1; ACSS2, acetyl-CoA synthetase 2; DLAT, dihydrolipoamide S-acetyltransferase; GPR43, G protein-coupled receptor 43; ILC3, type 3 innate lymphoid cell; MCT1, monocarboxylate transporter 1; PD-1, programmed death-1; PD-L1, programmed death-ligand 1; SMCT1, sodium-coupled monocarboxylate transporter 1; TCA cycle, tricarboxylic acid cycle; TME, tumor microenvironment.

In comparison, the protumorigenic effects of acetate involve broader metabolic reprogramming and epigenetic regulation. Under metabolic stress, cancer cells efficiently take up acetate via monocarboxylate transporter 1 (MCT1) or sodium-coupled monocarboxylate transporter 1 (SMCT1) 28, which is then converted into acetyl-CoA by mitochondria-localized acetyl-CoA synthetase 1 (ACSS1) or nucleocytosol-localized acetyl-CoA synthetase 2 (ACSS2) 42,43. This metabolic conversion serves multiple functions: energy supply (via the tricarboxylic acid cycle generating ATP), biosynthetic support (direct involvement in lipid synthesis), and histone acetylation promotion. Under hypoxic or glucose-limited conditions, acetate metabolism can account for up to 50% of the energy metabolism in glioblastoma (GBM), becoming a crucial alternative pathway for maintaining tumor energy homeostasis 39. In the cytoplasm, acetate serves as a direct carbon source for fatty acid synthesis, thereby supporting tumor growth 44. Furthermore, In low-oxygen or low-fat conditions, acetate uptake enhances the acetylation of lipid synthesis-related genes (*ACACA* and *FASN*), thus promoting lipid biosynthesis and aiding cancer cell survival and growth 45. Under glucose limitation, acetate increases histone H3K27 acetylation, promoting the expression of SNAI1, facilitating renal cancer cell migration 46. Recent studies have revealed that the metabolic reprogramming role of acetate in the TME is even more complex. In acidic TMEs, acetate does not regulate the expression of lipid synthesis genes (*SREBF1*, *FASN*, and *ACACA*) or total lipid content. Instead, it induces acetylation of the transcription factor SP1, alters polyamine metabolism, and enables pancreatic cancer cells to survive via the ACSS2-SP1-SAT1 axis 47. Protein O-linked N-acetylglucosamine (O-GlcNAc) modification acts as an intracellular nutrient sensor, and high-glucose diets promote cancer progression by inducing O-GlcNAc modification 48-50. As mentioned earlier, glucose is metabolized by the gut microbiome into acetate, and high-glucose diets increase acetate levels in the portal vein of HCC mouse models. After uptake by tumor cells, acetate upregulates glutamine and UDP-GlcNAc levels, enhancing the O-GlcNAc modification of eukaryotic elongation factor 1A1 (eEF1A1), thereby promoting tumor growth 23. Furthermore, acetate produced by glucose metabolism in tumor cells is secreted into the TME, upregulating the expression of monoamine oxidase B (MAO-B) and MCT1, which stimulates reactive astrocyte proliferation. The proliferation of reactive astrocytes is closely associated with poor prognosis in glioblastoma patients 34,51 (Figure 2).

In the studies described above, acetate exhibits a dual role in tumors. It suppresses tumor progression in NAFLD-HCC and CRC 38,40,41, while promoting growth in other cancers, including GBM, renal cancer, and pancreatic cancer 39,46,47, suggesting that its dual effects may be attributed to tumor heterogeneity. Even within the same tumor type, acetate can exert opposite effects: it inhibits tumor growth in NAFLD-HCC but promotes progression in DEN+CCl_4_-induced HCC models 23,38. The distinct effects of acetate may be associated with distinct metabolic patterns of the two aforementioned mouse models. Specifically, acetate may possess antitumor potential in lipid metabolism active NAFLD-HCC, while in glucose metabolism dominant HCC, it may function as a pro-tumor factor. Cellular stemness also influences the effects of acetate. Mashimo et al. reported that acetate, as an energy source, promotes the growth of GBM tumors 39. In contrast, Long et al. found that acetate inhibits the proliferation of glioblastoma stem-like cells (GSCs) derived from GBM 52. In animal models, GBM tumors generate energy by oxidizing acetate, supporting tumor growth. However, GSCs are more sensitive to acetate-induced epigenetic changes, which may interfere with their self-renewal capacity and suppress their proliferation. Furthermore, although acetate promotes tumor cell apoptosis in CRC 40,41, it reverses the anticancer effects of PINK1 overexpression in a mouse model, leading to tumor growth restoration 53. This suggests that the effects of acetate are regulated by genetic background. PINK1 overexpression activates mitophagy and suppresses the production of acetyl-CoA in the glycolytic pathway. Acetate, on the other hand, supplements acetyl-CoA, partially counteracting the metabolic changes induced by PINK1 overexpression, thereby attenuating its tumor-suppressive effects. Collectively, these results suggest that the ultimate effect of acetate in tumors largely depends on the intrinsic characteristics of tumor cells.

### 2.3 Acetate and Tumor Immunity

Tumor immunity refers to the process by which the immune system recognizes and eliminates tumor cells. However, this process is frequently suppressed by the tumor and the TME 54. Acetate exerts complex effects on tumor immunity. On the one hand, acetate is utilized by cancer cells to reduce their immunogenicity, thereby facilitating immune evasion. Studies have shown that acetate uptake via MCT1 promotes c-Myc acetylation, thereby enhancing Programmed death-ligand 1 (PD-L1) expression and subsequently suppressing the cytotoxic activity of immune cells against tumors 55. On the other hand, acetate enhances the antitumor capacity of CD8^+^ T cells. Under glucose-limited conditions, acetate boosts metabolic activity in CD8^+^ T cells, promoting CD3/CD28 stimulation-induced degranulation and effector functions 56. Furthermore, acetate increases histone acetylation and chromatin accessibility in an ACSS-dependent manner, enhancing transcription of the interferon gamma (IFN-γ) and cytokine production 57. In co-culture systems with cancer cells, acetate restores acetyl-CoA pools in CD8^+^ T cells, further amplifying their activity and IFN-γ secretion 58. However, within TME, cancer cells typically outcompete CD8^+^ T cells in acetate uptake and utilization due to their elevated expression of ACSS2 and MCT1 59,55. To overcome this metabolic competition and bolster CD8^+^ T cell immunity, several strategies have been explored: Depleting or inhibiting ACSS2 in cancer cells blocks acetate utilization, redirecting acetate to CD8^+^ T cells and enhancing their antitumor function 57. In addition, upregulating ACSS1 expression in CD8^+^ T cells improves their acetate metabolic capacity, thereby boosting antitumor activity 60. Alternatively, direct acetate supplementation elevates acetate levels in the TME, enhancing CD8^+^ T cell infiltration and IFN-γ production 58,61.

Furthermore, acetate also regulates other immune cells within the TME. In HCC, tumor-derived type 3 innate lymphoid cells (ILC3s) promote hepatic stellate cell (HSC) activation via an IL-17A-dependent pathway, driving liver fibrosis, which is associated with poor patient prognosis 62,63. Hu et al. demonstrated that acetate inhibits histone deacetylase activity and increases acetylation of SRY-box transcription factor 13 (Sox13) at the Lys30, thereby reducing IL-17A production in tumor-derived ILC3s and suppressing HCC progression 62. In the TME, M1 macrophages exert antitumor and immune-activating effects by secreting pro-inflammatory cytokines such as IL-6, IL-12, and TNF. In the meantime, M1 macrophages also play a crucial role in influencing the number and function of tumor-infiltrating CD8^+^ T cells 64,65. Acetate enhances transcription of acetyl-CoA carboxylase 1 (ACC1) by increasing histone acetylation at the ACC1 promoter and induces M1 macrophage polarization, thereby enhancing CD8^+^ T cell antitumor activity in HCC patients 66. Myeloid-derived suppressor cells (MDSCs), a heterogeneous myeloid cell population linked to chronic inflammation, exhibit potent immunosuppressive properties 67,68. In lung adenocarcinoma (LUAD), acetate activates the Gαq/calcium/PPAR-γ/Arg1 signaling pathway via free fatty acid receptor 2 (FFAR2), enhancing MDSC-mediated immunosuppression and facilitating tumor immune evasion 69 (Figure 2).

Acetate also exhibits a dual role in antitumor immunity, which is regulated by multiple factors. First, the type of target cells determines the effect of acetate: when acting on CD8⁺ T cells, ILC3s, or macrophages, acetate can enhance immune responses and promote antitumor activity 58,61,66; conversely, when acting on MDSCs, acetate can reinforce immunosuppressive signals, thereby facilitating tumor immune evasion 69. In addition, the source of acetate also influences its immunomodulatory effects: acetate derived from probiotics tends to promote antitumor immunity, whereas acetate generated through host metabolism is more likely to suppress immune responses. This difference may reflect variations in the site of production and the local microenvironment. In HCC, acetate is largely enriched in the liver via the gut-liver axis, where it can modulate immune cells within a relatively immune-tolerant microenvironment to enhance antitumor effects 62. In contrast, in tumors such as LUAD, characterized by hypoxia and lactate accumulation, cancer cell-derived acetate is more readily utilized as a metabolic substrate and can activate immunosuppressive pathways, thereby promoting tumor immune evasion 69. Overall, the immunomodulatory effects of acetate result from the interplay among its source, the local microenvironment, and the target cell type.

## 3. Propionate

### 3.1 Sources of Propionate

**Figure 3:**
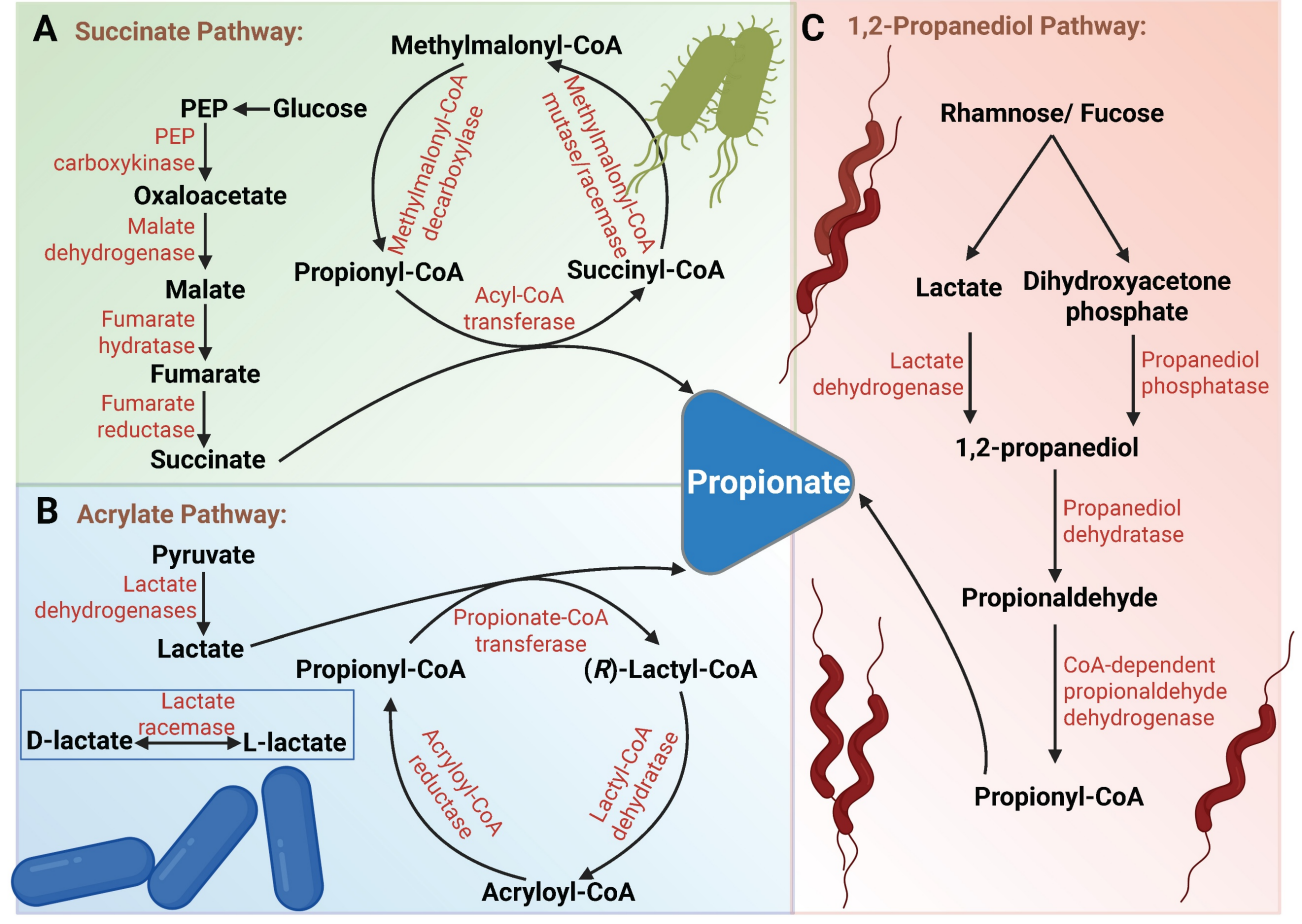
** Schematic illustration of propionate production by gut microbiota through fermentation of non-digestible carbohydrates.** (A) Succinate Pathway: Glucose is metabolized through glycolysis to PEP, which is carboxylated to oxaloacetate by PEP carboxykinase. Oxaloacetate is then reduced to malate by malate dehydrogenase. Malate undergoes dehydration to form fumarate, which is subsequently reduced to succinate by membrane-bound fumarate reductase. Succinate is then further metabolized to propionate via the methylmalonyl-CoA pathway. (B) Acrylate Pathway: Pyruvate is reduced to lactate by lactate dehydrogenase (NAD-dependent). Notably, both L-lactate and D-lactate, as enantiomers, are present in microbial metabolism, and their interconversion is facilitated by lactate racemase. Lactate is then conjugated with coenzyme A by propionyl-CoA transferase to form lactyl-CoA. This intermediate undergoes reversible syn-dehydration catalyzed by an oxygen-sensitive dehydratase, resulting in the formation of acryloyl-CoA. Acryloyl-CoA is ultimately reduced to propionyl-CoA by acryloyl-CoA reductase, followed by decarboxylation via propionyl-CoA transferase to generate propionate. (C) 1,2-Propanediol Pathway: Rhamnose and fucose are catabolized by deoxy sugar lyases to produce lactate and DHAP. Lactate is reduced to 1,2-propanediol by lactate dehydrogenase, while DHAP is dephosphorylated to 1,2-propanediol by propanediol phosphatase. 1,2-propanediol is then dehydrated by propanediol dehydratase to form propionaldehyde, which is subsequently oxidized to propionyl-CoA by CoA-dependent propionaldehyde dehydrogenase. Finally, propionate is released from propionyl-CoA via catalysis by propionate kinase. Abbreviations: DHAP, dihydroxyacetone phosphate; PEP, phosphoenolpyruvate.

Propionate in humans primarily originates from fermentation by gut microbiota. Glucose and lactate serve as the main fermentative substrates, which are converted into propionate through three major pathways (Figure 3). (1) The genus *Bacteroides* can produce propionate via the succinate pathway using methylmalonyl-CoA 70. (2) Soil bacterium *Clostridium propionicum* and *Negativicutes*, such as *Megasphaera elsdenii* in the rumen, can produce propionate via the acrylate pathway 71,72. The succinate pathway and acrylate pathway can be distinguished by incubating with isotopically labeled substrates 73,74. (3) Deoxysugars such as rhamnose can be converted into 1,2-propanediol through dihydroxyacetone phosphate or lactate, which is further metabolized to produce propionate. In the human gut, the anaerobic bacterium *Roseburia inulinivorans* has been found to generate propionate from fucose via the propanediol pathway 75,76. The associations between the microbiome and cancer have been extensively studied. In this section, we will focus on the role and regulatory mechanisms of microbe-derived propionate within the TME.

### 3.2 Propionate and Tumors

**Figure 4:**
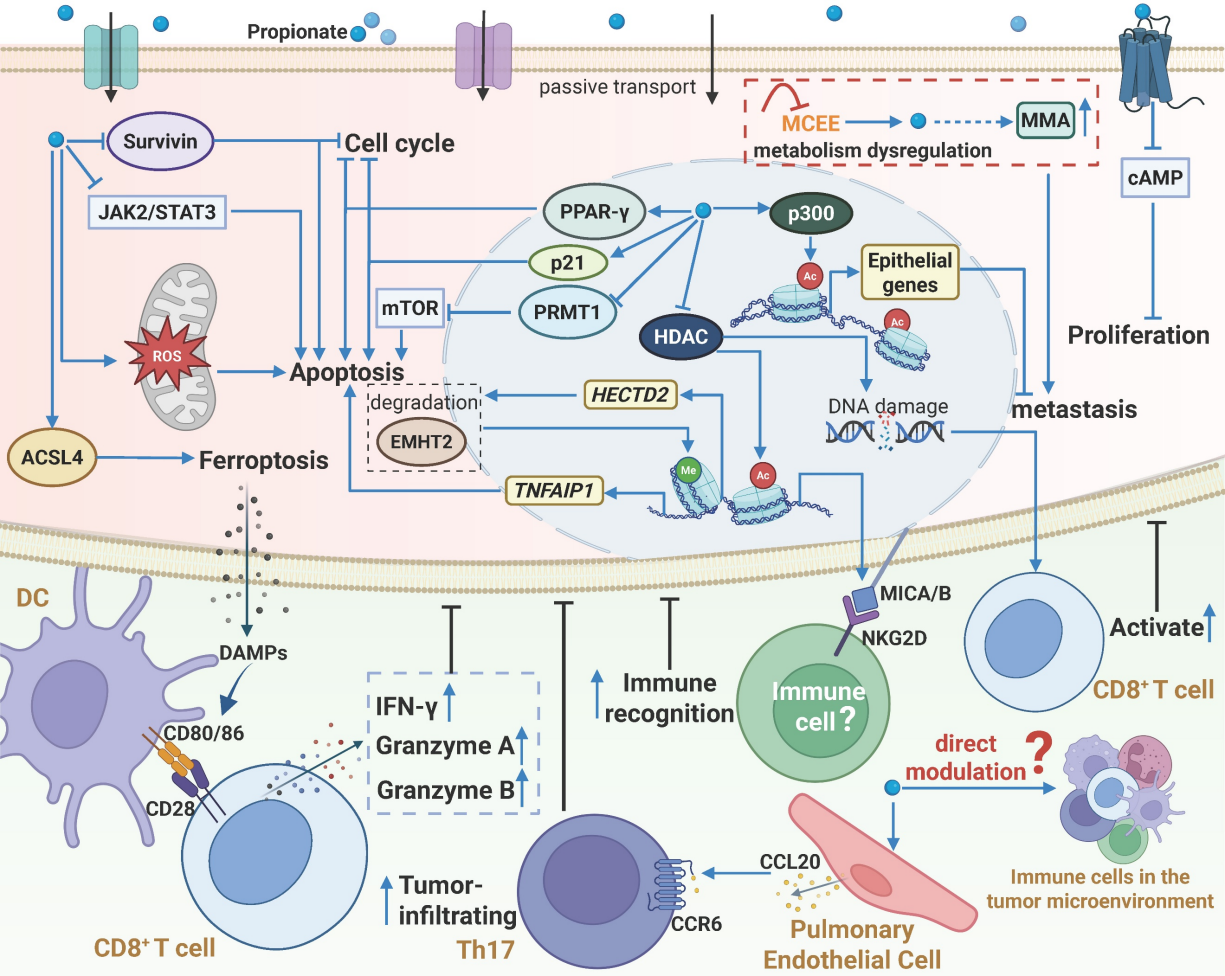
** The biological functions and mechanisms of propionate in the TME.** Similar to acetate, propionate plays a significant role in the TME through various mechanisms. Extracellular propionate can activate the GPR43 receptor, triggering a signaling cascade. Additionally, propionate enters cells, acting as an HDAC inhibitor or activating p300, which increases histone acetylation and gene transcription activity, accompanied by DNA damage. Propionate induces ROS generation or directly modulates multiple signaling pathways, leading to cell cycle arrest, inhibition of cancer cell proliferation and metastasis, and induction of apoptosis or ferroptosis. Moreover, propionate enhances the immunogenicity of cancer cells, thereby improving the recognition and killing ability of immune cells against cancer cells. Furthermore, propionate promotes pulmonary endothelial cells to secrete CCL20, recruiting Th17 cells to exert antitumor effects. However, there is currently no direct evidence that propionate regulates the function of immune cells in the TME. Notably, metabolic reprogramming in cancer cells inhibits MCEE activity, reducing propionate-driven anaerobic metabolism and increasing the production of MMA, which enhances the metastatic potential of tumors. Abbreviations: DC, dendritic cell; HDAC, histone deacetylase; MCEE, methylmalonyl-CoA epimerase; MMA, methylmalonic acid; ROS, reactive oxygen species; TME, tumor microenvironment.

In comparison to acetate, propionate generally inhibits tumor progression (Figure 4). First, propionate regulates a series of molecular events to induce cancer cell death 77-80. For example, propionate induces ROS generation and disrupts redox homeostasis, leading to mitochondrial fission, mitophagy, and subsequent ferroptosis and apoptosis 81,82. Furthermore, propionate, as a histone deacetylase (HDAC) inhibitor, blocks histone deacetylation, leading to chromatin relaxation and the transcriptional activation of pro-apoptotic genes such as HECT domain E3 ubiquitin protein ligase 2 (*HECTD2*), ultimately inducing apoptosis in cancer cells. Specifically, propionate upregulates HECTD2, promotes the degradation of euchromatic histone-lysine N-methyltransferase 2 (EHMT2), and reduces H3K9me2 levels in the tumor necrosis factor alpha-induced protein 1 (TNFAIP1) promoter region, thereby increasing TNFAIP1 expression and inducing apoptosis in CRC cells 83. Additionally, propionate suppresses cancer cell metastasis by activating the histone acetyltransferase p300, which mediates H3K27 acetylation and H3K4 methylation. This upregulates the transcription of epithelial genes, enhances intercellular contact and adhesion, and inhibits epithelial-to-mesenchymal transition (EMT), thereby impairing cancer cell migration and invasion 84. Notably, activation of p300 by propionate represents a novel mechanism distinct from its classical HDAC inhibition, revealing an unrecognized pathway for histone acetyltransferase (HAT) activation by propionate 85. Recent studies have found that propionate, by being converted to propionyl-CoA and modifying histones (such as H3K18pr and H4K12pr), significantly enhances chromatin accessibility, leading to the dysregulation of key cancer-associated genes, *MYC*, *JUN*, and *AHNAK2*, in CRC, thereby exhibiting antitumor effects 86. However, in breast and lung cancers, ERK2 activation shifts the role of propionate from antitumor to pro-tumor via metabolic dysregulation. Specifically, ERK2 inhibits methylmalonyl-CoA epimerase (MCEE), reducing propionate-driven anaplerosis and increasing methylmalonic acid (MMA) production 87,88. MMA accumulation in the TME then promotes tumor metastasis 89,90.

### 3.3 Propionate and Tumor Immunity

Beyond direct tumor suppression, propionate enhances cancer cell immunogenicity to activate antitumor immunity (Figure 4). Mowat et al. demonstrated that propionate directly stimulates CRC cells to enhance cytotoxic CD8^+^ T cell activation. Mechanistically, propionate inhibits histone deacetylation, causing DNA damage and upregulating chemokines, MHC-I, and antigen presentation genes in CRC cells. A feedback loop occurs: activated CD8^+^ T cells secrete IFN-γ, further stimulating cancer cells to amplify MHC-I expression and T cell activation. This loop is stronger in cancers with DNA mismatch repair defects and genomic instability 91. NKG2D, an immune-activating receptor on NK and effector T cells, enhances cytotoxicity when its ligands are expressed 92. In colon cancer, propionate induces metabolic reprogramming and histone acetylation/propionylation, leading to the upregulation of surface NKG2D ligands (MICA/B) on cancer cells, which activate immune responses 93. Propionate also triggers immunogenic cell death (ICD) in acute myeloid leukemia (AML). Concretely, propionate enhances ACSL4-mediated mitophagy, thereby releasing DAMPs to promote dendritic cell (DC) maturation and antigen presentation^81^. In melanoma lung metastasis models, propionate upregulates CCL20 in pulmonary endothelial cells, recruiting Th17 cells via the CCL20/CCR6 axis to reduce metastasis 94. However, the mechanism of CCL20 regulation remains unclear. Collectively, propionate shows broad potential in modulating cancer immunogenicity. In addition, studies have found that propionate can directly regulate γδ T cells 95, B cells 96, Tregs 97,98, and DCs 99 in other diseases 100. However, how it regulates immune cell function in the TME requires further exploration.

## 4. Butyrate

### 4.1 Sources of Butyrate

**Figure 5:**
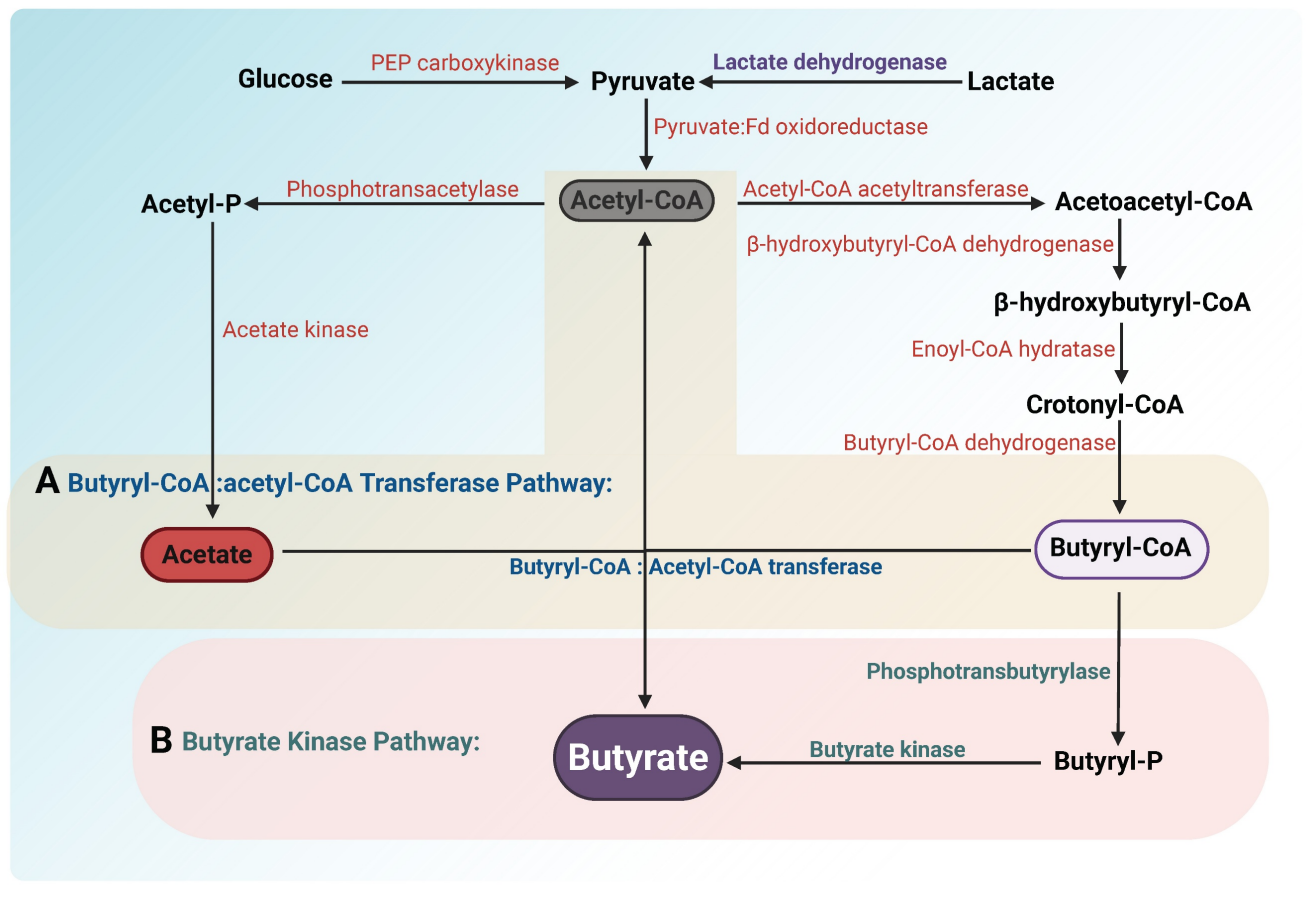
** Pathways of butyrate production by gut microbiota.** Glucose and lactate are converted into pyruvate through glycolysis and oxidation, respectively. Pyruvate is converted into acetyl-CoA by the enzyme pyruvate: ferredoxin oxidoreductase. Acetyl-CoA is subsequently condensed into acetoacetyl-CoA via acetyl-CoA acetyltransferase. Acetoacetyl-CoA is reduced to β-hydroxybutyryl-CoA by β-hydroxybutyryl-CoA dehydrogenase. This intermediate undergoes dehydration by enoyl-CoA hydratase to form crotonyl-CoA, which is then reduced to butyryl-CoA by butyryl-CoA dehydrogenase. Two distinct pathways are involved in the conversion of butyryl-CoA to butyrate: (A) Butyryl-CoA:acetate CoA-transferase pathway: In this pathway, butyryl-CoA is converted into butyrate through the action of butyryl-CoA:acetate CoA-transferase, which transfers the CoA group to acetate, producing butyrate and releasing acetyl-CoA. (B) Butyrate kinase pathway: Butyryl-CoA is converted to butyryl-P by phosphotransbutyrylase. Subsequently, butyrate is generated by butyrate kinase. Unlike the butyryl-CoA:acetate CoA-transferase pathway, this pathway requires the conversion of butyryl-CoA to butyryl phosphate, but instead does not consume acetate to generate butyrate.

Similar to propionate, butyrate in the human body is primarily synthesized through microbial metabolic activity. Microorganisms metabolize carbohydrates into pyruvate, which is converted to acetyl-CoA via the pyruvate dehydrogenase complex. Acetyl-CoA undergoes a series of enzymatic reactions to generate butyryl-CoA. Butyrate-producing bacteria then catalyze the conversion of butyryl-CoA to butyrate through two distinct pathways (Figure 5): (1) Phosphotransbutyrylase Pathway: Bacteria such as *Coprococcus comes* utilize butyryl-CoA:phosphate butyryltransferase and butyrate kinase to convert butyryl-CoA into butyrate 20,101. (2) Butyryl-CoA:Acetyl-CoA Transferase Pathway: Species like* Eubacterium rectale* and *Faecalibacterium prausnitzii* employ butyryl-CoA:acetyl-CoA transferase to produce butyrate, a process requiring acetate consumption 102,103. Notably, certain gut microbes (e.g., *Eubacterium hallii*, *Anaerostipes* spp.) can synthesize butyrate from both lactate and acetate, preventing lactate accumulation and maintaining intestinal homeostasis 20. Beyond gut microbiota, Intratumor bacteria such as *Roseburia* have also been reported to produce butyrate 104. As a key bacterial metabolite, butyrate not only plays a vital role in gut health but also exerts significant effects on tumorigenesis and progression 105. This section will elaborate on the mechanisms and implications of butyrate in cancer.

### 4.2 Butyrate and Cancer

**Figure 6:**
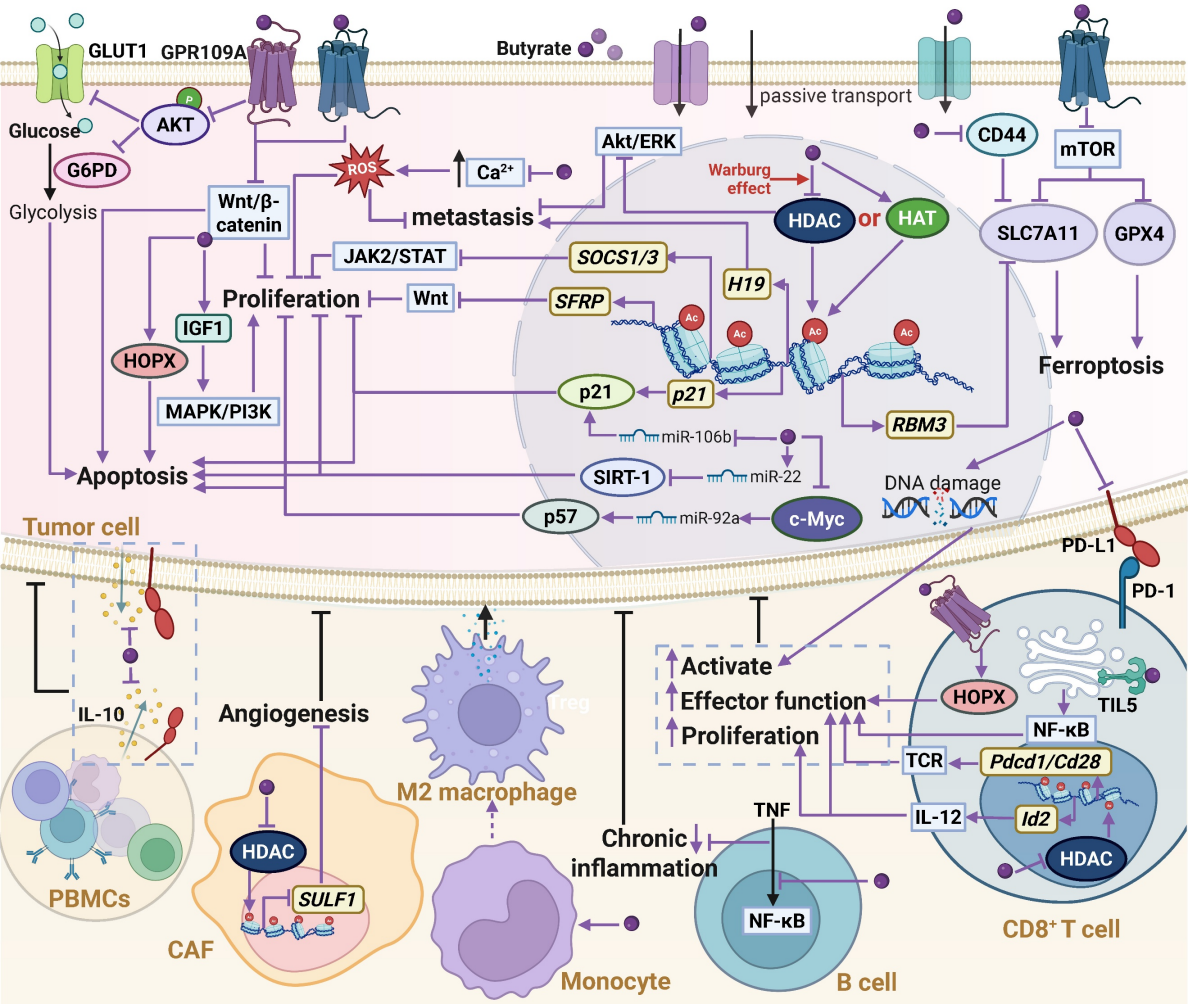
** The biological functions and mechanisms of butyrate in the TME.** Extracellular butyrate can activate GPR43 and GPR109A receptors to regulate downstream signaling and glucose metabolism, thereby suppressing cancer cell proliferation. Once inside the cell, butyrate functions as an HDAC inhibitor to increase histone acetylation, activate gene transcription, and modulate associated signaling pathways, leading to inhibition of proliferation and metastasis, while also inducing DNA damage, apoptosis, or ferroptosis. In addition, butyrate can regulate miRNA expression and influence gene regulatory networks; however, it may promote proliferation through the IGF1 pathway. Moreover, butyrate enhances antitumor immunity by increasing tumor cell immunogenicity and activating immune effector cells. Abbreviations: CAF, cancer-associated fibroblast; GLUT, glucose transporter 1; GPR109A, G protein-coupled receptor 109 A; HAT, histone acetyltransferase; HDAC, histone deacetylase; PBMCs, peripheral blood mononuclear cells; PD-1, programmed death-1; PD-L1, programmed death-ligand 1; ROS, reactive oxygen species; TME, tumor microenvironment.

Similar to acetate, butyrate exhibits a dual role in tumor initiation and progression (Figure 6), with its antitumor effects mediated through multiple mechanisms. As a signaling molecule, butyrate specifically activates G protein-coupled receptors (GPR41, GPR43, and the butyrate-specific receptor GPR109a) 106,107, thereby regulating cellular metabolism and key signaling pathways. In CRC, activation of the GPR109a-AKT axis by butyrate markedly reduces the membrane abundance of glucose transporter 1 (GLUT1) and glucose-6-phosphate dehydrogenase (G6PD), suppressing glucose uptake and glycolysis 108. Activation of GPR43 and GPR109a further inhibits the Wnt/β-catenin pathway, blocking proliferative signaling 109, while GPR43 activation also downregulates SLC7A11 and GPX4 in a cAMP-PKA-dependent manner, resulting in lipid peroxidation and ferroptosis in CRC cells 110. Within cells, butyrate functions as a HDAC inhibitor, increasing histone acetylation and thereby regulating gene expression and downstream signaling 111,112. For example, it enhances the acetylation of genes associated with calcium signaling, disrupting intracellular calcium homeostasis and inducing ROS generation, which inhibits proliferation and metastasis in HCC 113. HDAC inhibition by butyrate also modulates multiple pathways, including AKT/ERK and JAK2/STAT3, thereby suppressing invasion in CRC as well as proliferation and angiogenesis in myeloproliferative tumors 114,115. Regarding cell death, butyrate promotes apoptosis by upregulating pro-apoptotic proteins (Bax, p53) and downregulating Bcl-2 116,117, while also inducing ferroptosis in endometrial cancer, CRC, and PDAC cells through regulation of RBM3, CD44, and SLC7A11 expression balance as well as lipid metabolism 118-120. Moreover, the antitumor effects of butyrate involve miRNA-mediated regulation 121,122. In CRC, butyrate downregulates miR-106b, relieving repression of *p21*, leading to its overexpression and cell cycle arrest, thereby inhibiting cell proliferation 121. It also suppresses c-Myc to inhibit miR-92a transcription, increasing p57 expression and further suppressing proliferation while promoting apoptosis 123. In HCC, butyrate enhances miR-22 expression, inducing apoptosis and inhibiting cell proliferation 124.

However, the pro-tumorigenic effects of butyrate have also been reported. For example, microbiota-derived butyrate can promote colorectal carcinogenesis by inducing senescence of colonic epithelial cells and driving abnormal proliferation and transformation in mouse colon epithelium 125,126. This phenomenon, which contradicts the tumor-suppressive role of butyrate in CRC, is referred to as the “butyrate paradox”. Specifically, in normal colonic epithelial cells, butyrate is efficiently metabolized into acetyl-CoA, which enhances HAT activity and promotes cell proliferation. In contrast, in cancer cells, due to the Warburg effect, whereby cells preferentially undergo glycolysis rather than oxidative phosphorylation even in the presence of oxygen, butyrate metabolism is impaired, leading to its intracellular accumulation. Under these conditions, butyrate functions as a HDAC inhibitor to suppress tumor progression. Although both mechanisms ultimately increase histone acetylation, they target distinct sets of genes 127. Therefore, the differential metabolic preferences between normal colonic epithelial cells and CRC cells are considered as a key determinant driving the dual roles of butyrate. Moreover, In prostate cancer, butyrate has also been shown to elevate circulating insulin-like growth factor-1 (IGF-1) levels, thereby activating the MAPK/PI3K pathway and promoting tumor progression 128. The divergent outcomes observed in CRC, HCC, and prostate cancer are largely attributed to the dose-dependent effects of butyrate: at low concentrations, butyrate tends to promote tumor progression, whereas at high concentrations, it exerts inhibitory effects 129-131. Because butyrate is extensively consumed in the intestine and liver, its systemic bioavailability is markedly reduced, resulting in relatively low concentrations within prostate tumors. Consequently, butyrate fails to accumulate intracellularly and instead acts as an extracellular signaling molecule that promotes tumor growth 128. Nevertheless, this dose dependency is not absolute; under conditions of dysbiosis, cholestasis, or inflammation, even high concentrations of butyrate have been reported to facilitate HCC progression 132. Therefore, the dual roles of butyrate cannot be explained by a single factor but are instead determined by the interplay among cellular metabolic states, local concentration gradients, and the host's physiological and pathological context.

### 4.3 Butyrate and Tumor Immunity

Butyrate is a key metabolite mediating crosstalk between the gut microbiome and the immune system. Many studies have reported that butyrate has the effect of promoting tumor immunity. In terms of immunogenic modulation, butyrate enhances CRC cells immunogenicity and potentiates their ability to activate CD8^+^ T cells 91,133. Concurrently, it directly amplifies CD8^+^ T cell function. In gastric cancer(GC), butyrate acts as a GPR109A agonist and amplifies the cytotoxicity of CD8^+^ T cells and CAR-Claudin 18.2^+^ CD8^+^ T cells via the GPR109A/HOPX axis 134. Interestingly, studies report that butyrate does not alter GPR41/43/109A expression 135, and GPR blockade fails to abrogate the effects of butyrate on CD8^+^ T cells 136, suggesting a GPR-independent mechanism. Kang et al. further identified TLR5 as a novel butyrate receptor, whose upregulation on CD8^+^ T cells activates the NF-κB pathway, enhancing cytotoxicity 135. Additionally, butyrate modulates CD8^+^ T cells via HDAC inhibition. In B16-F0-bearing mice, it elevates H3K27ac at the *Pdcd1* and *Cd28* promoters in CD8^+^ T and Vδ2^+^ T cells, enhancing antitumor cytokine production through T-cell receptor (TCR) signaling pathway 137. Butyrate also induces ID2 expression and enhances IL-12 signaling, thereby boosting cytotoxic CD8^+^ T cell responses 136.

Beyond T cells, butyrate augments NK cell activity and promotes liver-resident NK cell development 138. Moreover, it activates macrophages to reinforce the intestinal mucus barrier 139 and suppresses DC antigen presentation, reducing pro-inflammatory cytokine release 140,141. These findings suggest that butyrate has regulatory effects on innate immunity. For instance, in advanced GC patients, it was found that butyrate can reduce the expression of immunosuppressive factors PD-L1 and IL-10 in peripheral blood mononuclear cells (PBMCs), exerting antitumor effects 142. Additionally, some butyrate-producing bacteria exhibit antitumor activity via butyrate secretion. *Eubacterium rectale* is a beneficial microbiota that helps prevent primary intestinal lymphoma. It produces butyrate to suppress the TNF/TLR4/MyD88 signaling pathway and inhibit the NF-κB pathway in B cells, thus reducing TNF-associated intestinal inflammation and the incidence of lymphoma in *Eμ-Myc* mice 143. Despite the large body of research supporting the role of butyrate in promoting immunity in the TME, other studies reported its immunosuppressive effects. For example, in a lung cancer (LC) relapse model, butyrate derived from *Roseburia* inhibited HDAC2, increased H3K27 acetylation at the H19 promoter, and induced M2 macrophage polarization, thereby increasing the expression of H19 in tumor cells and promoting lung cancer metastasis 104. In addition to regulating immune cells in TME, butyrate also modulates cancer-associated fibroblasts (CAFs), contributing to its antitumor effects. Specifically, Wang et al. identified a subset of CAFs expressing high levels of sulfatase 1 (SULF1), which is associated with poor prognosis in CRC patients. Butyrate suppresses SULF1 expression in CAFs by inhibiting HDAC activity, thereby attenuating SULF1-mediated angiogenesis 144 (Figure 6).

In contrast to its effects on tumor cells, butyrate enhances CD8⁺ T cell function across multiple tumor types. This effect is observed not only in the butyrate-rich intestinal environment but also in subcutaneous tumors with relatively low concentrations of butyrate, suggesting a broadly conserved regulatory role, albeit through distinct mechanisms 135,136. Comparatively, its modulation of innate immune cells appears to be more tissue-specific: in the stomach and intestine, butyrate promotes antitumor immunity by acting on PBMCs and B cells 142,143, whereas in the lung it drives macrophage polarization toward an immunosuppressive phenotype, thereby attenuating antitumor responses. Such differential effects may be attributed to the lower butyrate concentrations in distal tissues 104. Moreover, butyrate can inhibit angiogenesis by regulating CAF activity, further contributing to its antitumor properties 144. Collectively, these findings highlight the cell-specific and tissue-dependent roles of butyrate in the regulation of tumor immunity.

## 5. Other SCFAs

### 5.1 Formate

Within the TME, formate originates not only from microbial secretion but also from host cell metabolism, primarily through the conversion of cellular serine mediated by serine hydroxymethyltransferase (SHMT) and methylenetetrahydrofolate dehydrogenase (MTHFD) 145. As a key regulator of tumor metastasis, formate directly promotes migration and invasion in cancer cell lines 146-148. For instance, formate derived from *Fusobacterium nucleatum* can activate aryl hydrocarbon receptor (AhR) signaling, promoting CRC invasion and enhancing cancer stemness 149. Formate also reprograms lipid metabolism in glioblastoma cells, facilitating an invasive phenotype through MMP-mediated degradation of extracellular matrix (ECM) proteins 150. Furthermore, as a nucleotide synthesis precursor, formate fuels purine and pyrimidine production to meet the high nucleotide demands of cancer cells 151. In PDAC, cancer cells generate formate via an IDO1-dependent pathway. This formate enters the tetrahydrofolate (THF) cycle, where it is co-utilized by cancer cells and pancreatic stellate cells for purine nucleotide synthesis, driving proliferation 152. Formate also modulates immune responses. Recent studies have shown that in melanoma, formate can enhance CD8⁺ T cell-mediated antitumor immunity via activation of the Nrf2 pathway 148. In contrast, in CRC, formate promotes the expansion of Th17 cells in the mesenteric lymph nodes (MLN) and increases the release of pro-inflammatory cytokines, thereby accelerating tumor initiation and progression 149. Overall, formate drives tumor progression by reprogramming cancer metabolism and perturbing immune regulation. Notably, with respect to CD8⁺ T cells, various SCFAs generally enhance their proliferation, activation, and cytotoxic function, thereby exerting broad antitumor immune effects.

### 5.2 Valerate

As a short-chain fatty acid with five carbon atoms, valerate predominantly exhibits antitumor properties within the TME. Lau et al. demonstrated that *Lactobacillus acidophilus*-derived valerate suppresses NAFLD-HCC development and enhances intestinal barrier integrity. Mechanistically, valerate binds to hepatocyte surface receptors GPR41/43, inhibiting the Rho-GTPase pathway and suppressing NAFLD-HCC initiation 153. Valerate also inhibits proliferation of breast and liver cancer cells via epigenetic regulation 154,155, including HDAC inhibition and DNA methylation pattern alterations. Previous studies have confirmed its potent anti-inflammatory effects in inflammatory and autoimmune diseases 156, suggesting a potential role in modulating tumor immunity within the TME. *In vitro*, treatment of cytotoxic T lymphocytes (CTLs) with valerate enhances the expression of effector molecules (e.g., CD25, IFN-γ, TNF-α) through HDAC inhibition, thereby boosting their antitumor activity 25.

### 5.3 Isobutyrate and Isovalerate

Unlike straight-chain SCFAs, the formation of branched-chain SCFAs (such as isobutyrate and isovalerate) primarily depends on the metabolism of undigested proteins by gut microbiota 157. These metabolites are present at relatively low concentrations in the body, and research on their regulatory effects on tumors has been limited. Notably, isobutyrate and isovalerate have been found to exhibit similar HDAC inhibitory effects on cancer cells 158. Recent studies have demonstrated that isobutyrate exerts potent antitumor effects by modulating immune cell activity and tumor growth. Mechanistically, isobutyrate reduces the expression of programmed death-1 (PD-1) in T cells, increases the expression of MHC class II receptor HLA-DR, and activates T cells. Oral administration of isobutyrate enhances the antitumor effects of anti-PD-1 antibodies, reducing tumor volume and increasing the number of tumor-infiltrating T cells 159. These findings highlight the potential of branched-chain short-chain fatty acids in tumor regulation, and further research into these metabolites will help us gain a more comprehensive understanding of the role of SCFAs in tumor progression.

## 6. SCFAs and Cancer Treatment

### 6.1 Dietary, Microbiota, and Metabolic Interventions Targeting SCFAs

Clinical studies demonstrate that cancer patients exhibit dysregulated levels of SCFA-producing microbiota and SCFAs compared to healthy individuals (Table 1). Microbes and SCFAs that are downregulated in cancer or upregulated after treatment are generally considered beneficial 160-162 (e.g.,* Bifidobacterium pseudolongum* 38, *Roseburia intestinalis* 135, *Lactobacillus acidophilus* 153). Therapeutic strategies to restore beneficial SCFAs include SCFA supplementation 163,164, fecal microbiota transplantation (FMT) 62,134,165, high-fiber diets 166, and probiotics 109,167. Certain probiotics and bioactive metabolites described above have shown significant therapeutic effects in both clinical and preclinical studies. For instance, acetate produced by *Bifidobacterium pseudolongum* significantly downregulates progression in the NAFLD-HCC mouse model. Administration of *Bifidobacterium pseudolongum* or acetate can significantly inhibit NAFLD-HCC progression 38. Likewise, FMT from high-SCFA donors significantly reduces tumor burden in HCC mice 62. A clinical trial found that Alaska Native (AN) people have an increased risk of CRC due to a deficiency in colonic butyrate, caused by low dietary fiber intake. Supplementation with high-dose soluble fiber was shown to significantly reduce cancer risk in AN people (NCT03028831). Similarly, other clinical studies have reported comparable findings 168,169. In CRC patients, oral administration of SCFA-producing probiotics or prebiotics-encapsulated probiotic spores can modulate the gut microbiota, increase the abundance of beneficial microorganisms, and significantly reduce the abundance of pathogenic microbes associated with CRC, highlighting the potential therapeutic benefits of probiotics in CRC treatment (NCT03072641) 170.

Conversely, microbes and SCFAs that are upregulated in cancer (e.g., *Fusobacterium nucleatum* 149, *Rikenellaceae*, *Clostridiales* 171) are often pathogenic 69,104,172,173. Therapeutic approaches targeting these harmful factors primarily rely on antibiotics and metabolic enzyme inhibition. In a high-fat diet-induced prostate cancer mouse model, treatment with a broad-spectrum antibiotic cocktail (Abx) significantly reduced pathogenic bacterial abundance and fecal SCFA levels, thereby suppressing tumor growth 171. Blocking key metabolic pathways has also shown therapeutic promise. Inhibition of SHMT2/MTHFD1 to target formate metabolism induces a “folate trap” impeding nucleotide synthesis and promoting cancer cell death 174,175. For instance, in Burkitt lymphoma (BL) cells, SHMT2 inhibitors deplete intracellular formate and trigger apoptosis 175. Likewise, by inhibiting ACSS2 to block acetate utilization, the level of acetyl-CoA is reduced in cancer cells, thereby decreasing its ability for lipid synthesis and energy metabolism, effectively hindering tumor progression. In the preclinical breast cancer model, targeting ACSS2 using CRISPR-Cas9-guided editing or small molecule inhibitors (such as VY-3-135) to suppress acetate metabolism disrupts cancer cell adaptation to metabolic stress, leading to a significant reduction in tumor growth 56,176. Additionally, the first human clinical trial of ACSS2 inhibitors for cancer therapy is currently underway (NCT04990739). Overall, whether to supplement SCFAs or inhibit their production to suppress tumor progression depends on the specific cancer context. Overall, whether supplementing or inhibiting the production or metabolism of SCFAs to suppress tumor progression depends on the specific cancer context and the composition of the microbiome. In some cases, SCFAs may exert beneficial effects by reshaping the microbiome or improving the TME, whereas in other cases, they may promote tumor growth by facilitating metabolic reprogramming. Inter-individual differences in the microbiome may be a key factor in these response variations 177. Therefore, blindly supplementing SCFAs or inhibiting their production and metabolism may not consistently achieve the desired outcomes in clinical treatments.

### 6.2 SCFAs as Adjuvants in Radiation and Chemotherapy

**Figure 7:**
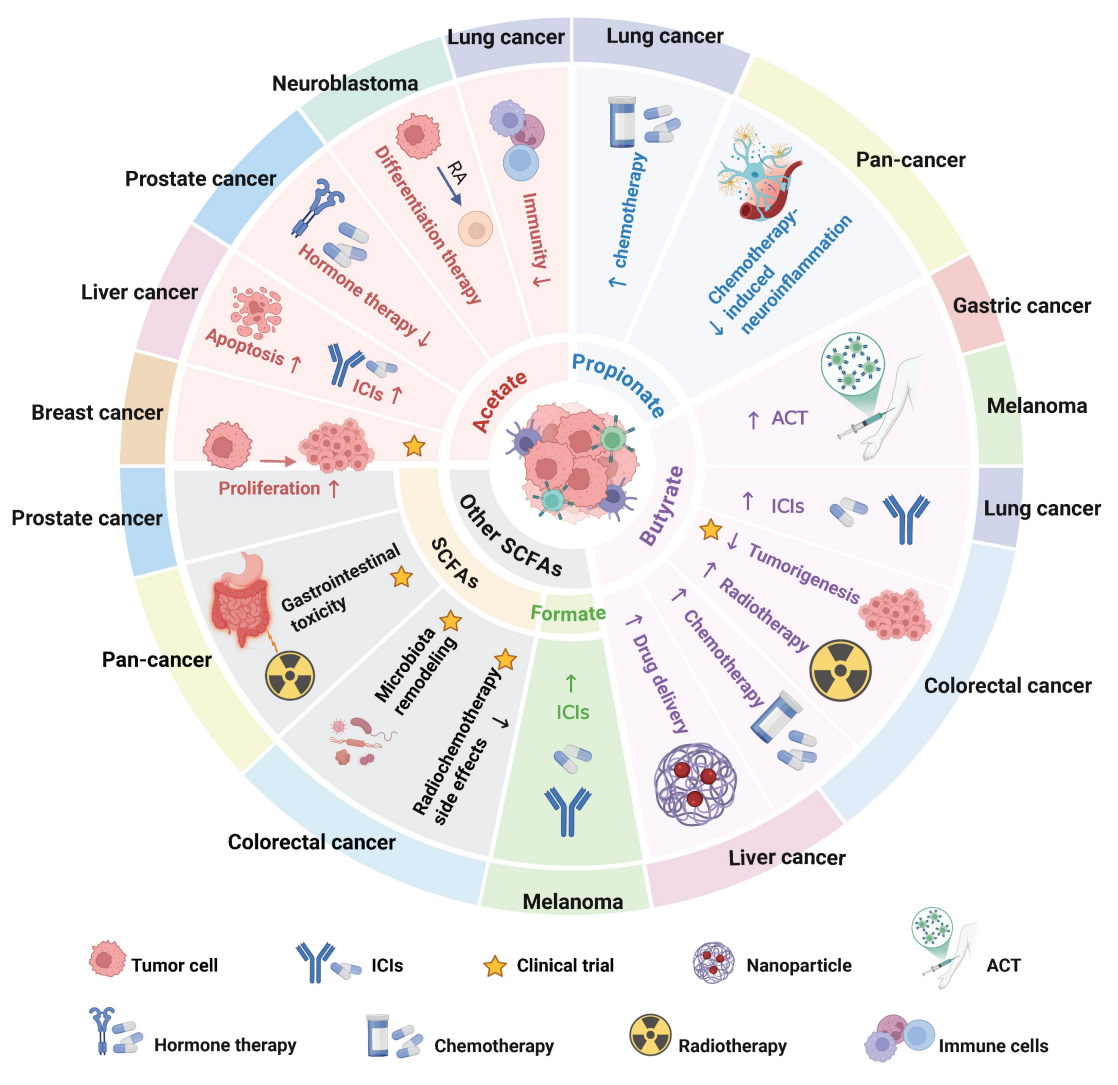
** Roles and clinical applications of SCFAs in various tumor types.** The roles of acetate, propionate, butyrate, and other SCFAs in various tumor types and their potential clinical applications, including adjunctive roles in chemoradiotherapy, immunotherapy, and other therapeutic modalities. Abbreviations: ACT, adoptive T cell therapy; ICIs, immune checkpoint inhibitors; RA, retinoic acid.

Increasing evidence suggests that SCFAs can enhance the efficacy of cancer radiotherapy and chemotherapy while mitigating treatment-related toxicities (Figure 7). For instance, butyrate produced by three probiotic strains, *Lactobacillus plantarum* S2, *L. pentosus* S3, and *L. rhamnosus* 14E4, enhances the sensitivity of doxorubicin-resistant CRC cell lines (HT29-dx) to the drug 178. Dong et al. demonstrated that FMT promotes the accumulation of *Roseburia intestinalis* in the gut, which secretes butyrate and activates radiation-induced autophagy via the OR51E1/RALB axis, thereby enhancing the radiotherapy sensitivity of CRC in mice 179. Clinically, serum butyrate levels are significantly higher in oxaliplatin (OXA)-responsive CRC patients compared with non-responsive individuals 136. Butyrate supplementation effectively overcomes resistance, resensitizing CRC cells to OXA 136,180,181. Additionally, in lung cancer models, resistance to cisplatin, a first-line chemotherapeutic agent, is commonly mediated by EMT 182. Treatment with propionate was found to upregulate epithelial transcriptional programs, strengthen cell-cell adhesion, and suppress chemoresistant EMT phenotypes, thereby sensitizing lung cancer cells to cisplatin 84. Meanwhile, studies have shown that butyrate can also enhance the therapeutic efficacy of sorafenib in HCC mouse model 113.

Moreover, SCFAs can alleviate the side effects caused by conventional therapies. In murine pancreatic cancer models, the combination of gemcitabine and butyrate not only significantly reduced cancer-associated stroma formation, but also preserved the integrity of the intestinal mucosal barrier, improved fecal microbiota composition, and alleviated chemotherapy-induced renal and hepatic injury 183. Similarly, SCFAs have been shown to mitigate 5-fluorouracil (5-FU)-induced neuroinflammation 184. Recent clinical studies revealed that lower plasma levels of SCFAs were significantly associated with more severe radiotherapy-related fatigue in patients with head and neck cancer 185. Moreover, SCFAs were demonstrated to reduce gastrointestinal complications and microbiota dysbiosis in CRC patients undergoing chemotherapy (ChiCTR2000040916) 186. Ongoing clinical studies are also investigating SCFA supplementation in patients receiving pelvic radiotherapy, with the aim of improving quality of life and reducing treatment-related toxicities (NCT04700527). Although SCFAs have shown potential in enhancing the efficacy of radiotherapy and chemotherapy while alleviating side effects, the current clinical studies have small sample sizes, and the assessment of dosage, safety, and individual variability remains insufficient. Excessive supplementation may lead to gut microbiota dysbiosis or other adverse effects. Therefore, large-scale clinical trials are needed in the future to systematically evaluate the safety and efficacy of SCFAs in chemotherapy and radiotherapy.

### 6.3 SCFAs as Adjuvants in Immunotherapy

As discussed in previous sections, SCFAs are recognized as effective modulators of immune responses, particularly in the context of cancer immunotherapy involving immune checkpoint inhibitors (ICIs) and adoptive T cell therapy (ACT) (Figure 7).

SCFAs have shown promising results in combination with ICIs. For example, in an HCC mouse model, treatment with PD-1 monoclonal antibody combined with acetate improved liver damage and inhibited tumor growth 62. In a CRC mouse model, oral butyrate administration three days after tumor inoculation, followed by combination with anti-PD-1 therapy, increased the number of tumor-infiltrating CD8^+^ T cells and the production of their effector cytokines, significantly inhibiting tumor growth 135. The enhancement of anti-PD-1 therapy by butyrate was also validated in an non-small cell lung cancer (NSCLC) mouse model 137. Interestingly, formate, a short-chain fatty acid that has been reported to promote cancer, combined with anti-PD-1 therapy, also enhanced CD8^+^ T cell-mediated tumor control in a B16-OVA tumor model and improved mouse survival 187. Additionally, Several clinical studies have shown that high concentrations of SCFAs in feces are significantly associated with longer progression-free survival and relatively higher response rates to PD-L1 inhibitors (ChiCTR2000032088) 162,188. However, the role of SCFAs in immunotherapy is not without controversy. In mouse melanoma models and metastatic melanoma patients, elevated serum levels of butyrate and propionate were associated with resistance to CTLA-4 blockade and a higher proportion of Treg cells. Furthermore, in melanoma-bearing mice, supplementation with butyrate combined with α-CTLA-4 inhibited dendritic cell maturation and the accumulation of effector and memory tumor-specific CTLs 189. Additionally, targeting the pro-cancer effects of acetate in lung adenocarcinoma and NSCLC by inhibiting acetate receptors (FFAR2) and downstream molecules regulated by acetate (USP10) slowed tumor growth and improved responses to ICI therapy 55,69. While many studies have reported the beneficial role of SCFAs in enhancing cancer treatment, other studies have also highlighted their anti-inflammatory properties 190. Therefore, the complex multifaceted effects of SCFAs are not surprising.

Given regulatory roles of SCFAs in T cell proliferation, cytotoxicity, and tumor infiltration, recent studies explore SCFAs as adjuvants for ACT. Prasad et al. found that SCFAs can enhance the metabolic adaptability of CAR-T cells, thereby improving their tumor-killing capacity 191. In parallel, Luu et al. demonstrated that *in vitro* treatment of CTLs and chimeric antigen receptor (CAR) T cells with butyrate or valerate enhanced mTOR-mediated metabolic sensing, inhibited class I HDAC activity, and upregulated effector molecule expression, thereby improving antitumor efficacy in melanoma and pancreatic cancer models 25. Similarly, Yu et al. reported that butyrate enhanced the cytotoxic activity of CD8^+^ T cells and CAR-Claudin 18.2^+^ CD8^+^ T cells against GC cells through GPR109A and homeobox protein HOPX signaling, as demonstrated by *in vitro* co-culture experiments and *in vivo* tumor-bearing mouse models 134. Current findings suggest that SCFAs enhance T cell functionality and therapeutic efficacy *in vivo*, underscoring their clinical potential as ACT adjuvants. However, available data also suggest that the effects of SCFAs may be counterproductive due to the complexity of the TME 192. Additionally, studies on SCFAs as adjuncts to ACT are still limited, underscoring the need for more extensive and in-depth research to fully explore their clinical potential.

### 6.4 Other Applications of SCFAs in Cancer Diagnosis and Therapy

Differentiation therapy is a treatment strategy that aims to alter the differentiation state of cancer cells using differentiation inducers, leading to the loss of malignant phenotypes. It has been successfully applied in the treatment of acute promyelocytic leukemia (APL) 193,194, although its efficacy in solid tumors has been less satisfactory 195. Notably, Li et al. suggested that in a mouse neuroblastoma model, combining acetate with differentiation therapy (retinoic acid induction) not only restored histone acetylation under hypoxic conditions, but also reestablished the expression of neuronal differentiation markers and neuronal differentiation morphology, significantly improving the *in vivo* efficacy of retinoic acid 196. Hormonal therapy regulates the body's endocrine balance to treat tumors, and it is considered a first-line treatment for prostate cancer. During prostate cancer treatment, acetate metabolism enhances c-MYC expression during neurodifferentiation (NED), thereby promoting resistance of primary adenocarcinoma prostate cancer (PCa) to hormonal therapy. Studies have shown that combining ACSS2 inhibitors with hormonal therapy can significantly enhance PCa sensitivity to the hormonal drug enzalutamide (ENZA), thereby effectively inhibiting tumor growth 197. Furthermore, butyrate can be used to modify nanoparticles to enhance drug efficacy. Research indicates that oral butyrate-modified nanoparticles effectively and persistently promote trans-epithelial transport in the gut, drug accumulation in the liver, and drug uptake by HCC cells, thereby enhancing liver cancer treatment 198. Additionally, acetate exhibits clinical potential in imaging technologies. ^11^C-acetate positron emission tomography (PET) has emerged as a promising imaging modality for prostate cancer diagnosis and therapeutic monitoring (NCT01144897) 34,199. The combined application of SCFAs in other cancer therapies offers new hope for treatment and diagnostic strategies (Figure 7). However, several limitations currently exist, including insufficient translational research, significant variability in efficacy across different cancer types and patient populations, and constraints in clinical applications. Specifically, ensuring the precise delivery and targeted therapy of SCFAs remains a challenge, and there is a lack of unified mechanistic understanding. Furthermore, the choice of different dosages, combination regimens, and administration methods in clinical trials can all influence treatment outcomes. In the future, with advancements in precise delivery technologies, deeper mechanistic studies, and rigorous clinical validation, these strategies are expected to be optimized and more effectively integrated into individualized cancer treatment plans.

## 7. Conclusions and Perspectives

SCFAs, as key functional metabolites within the TME, profoundly influence tumor initiation, progression, and therapeutic response by orchestrating metabolic reprogramming, epigenetic modifications, and immune microenvironmental dynamics. SCFAs exhibit a 'double-edged sword' effect in tumor regulation—capable of both suppressing tumor growth and promoting malignant phenotypes. This duality highlights the biological complexity of SCFAs and underscores the necessity of precise modulation based on tumor heterogeneity, host metabolic status, and microbiota composition.

From a metabolic perspective, SCFAs can fuel tumor growth and survival by supplying acetyl-CoA or one-carbon units to regulate energy metabolism, lipid biosynthesis, and nucleotide synthesis. Conversely, under certain conditions, SCFAs may induce oxidative stress, mitochondrial dysfunction, and lipid peroxidation, thereby triggering tumor cell death. This 'metabolic switch' effect is largely dependent on pH, oxygen availability, and nutrient competition within the TME, suggesting that future interventions should incorporate metabolic imaging techniques (e.g., ^11^C-acetate PET) to dynamically monitor tumor metabolic profiles and optimize SCFA-based therapeutic strategies 200. At the epigenetic level, SCFAs reshape chromatin accessibility and regulate gene expression by inhibiting HDACs, activating HATs, and providing acyl groups. These epigenetic modifications influence tumor cell proliferation, migration, and survival. Notably, the epigenetic effects of SCFAs are cell type-specific: their regulation of HDACs, HATs, and endogenous acyl donors differs between normal and cancer cells, leading to distinct target genes being modified and thereby producing divergent downstream phenotypes. This cell type-dependent regulation provides a theoretical rationale for developing precise combination therapies targeting epigenetic pathways. Immune regulation represents one of the key mechanisms by which SCFAs influence cancer therapy. On the one hand, SCFAs can enhance the metabolic adaptability and cytotoxicity of CD8⁺ T cells, promote M1 macrophage polarization, and modulate the function of ILCs to strengthen antitumor immunity. On the other hand, SCFAs may impair immune efficacy by activating regulatory Tregs or suppressing DCs antigen presentation. This bidirectional immunomodulation suggests that future studies should incorporate spatial profiling of immune cell subsets to delineate the spatiotemporal landscape of SCFA action within the TME, thereby enabling precision enhancement of immunotherapeutic outcomes 100,201.

As key metabolites of the gut microbiota, SCFAs exhibit substantial potential in tumor development and therapy, offering new avenues to improve cancer treatment outcomes. From a translational perspective, SCFAs have demonstrated value as adjuvants to chemotherapy, radiotherapy, and immunotherapy. Strategies such as increasing SCFA levels through dietary fiber, probiotics, or direct supplementation can enhance therapeutic sensitivity while alleviating toxic side effects. Moreover, targeting specific SCFA-metabolizing enzymes or receptors holds promise for reversing protumor metabolic phenotypes. However, the efficacy of SCFAs is constrained by concentration dependence, tissue specificity, and interindividual differences in gut microbiota, leading to considerable variability in therapeutic responses.

To overcome these limitations, future research should focus on three directions: (1) applying single-cell multi-omics to delineate the spatiotemporal networks and cell-type-specific effects of SCFAs; (2) developing precision delivery systems, such as engineered probiotics or nanoparticle-based carriers, to optimize tissue targeting and mitigate concentration dependence; and (3) tailoring SCFA-based interventions to individual metabolic and microbiota profiles, thereby advancing truly individualized cancer treatment.

## Figures and Tables

**Table 1:** Changes in SCFAs levels and their properties in cancer patients.

Cancer types	Compare groups	Levels of SCFAs and associated-bacteria	Properties	Ref.
CRC	CRC patients vs HCs (human fecal)	SCFAs↓	Beneficial	180
CRC	CRC patients vs HCs (human fecal)	Butyrate/*Roseburia intestinalis*↓	Beneficial	135
HCC	HCC patients vs HCs (human plasma)	Butyrate↓	Beneficial	113
HCC	Recurrence vs non-recurrence patients (human plasma/ fecal)	Acetate/*Bacteroides thetaiotaomicron*↓	Beneficial	66
GC	GC patients vs HCs (human fecal)	Butyrate↓	Beneficial	134
GC	GC patients vs HCs (human fecal)	Butyrate/*Faecalibacterium* and *Bifidobacterium*↓	Beneficial	142
BC	BC patients vs HCs (human fecal microbial compositions)	Propionate↓	Beneficial	202
BC	BC patients with depression vs those without depression (human plasma)	Acetate↓	Beneficial	58
AML	AML patients vs HCs (human fecal)	Propionate↓	Beneficial	81
NSCLC	Human functional genomic	Propionate metabolism↓	Beneficial	84
Multiple Myeloma	CRMM vs RRMM	Aropionate and butyrate/*Agathobacte*r↓	Beneficial	203
GC	GC vs IM vs CSG patients (human plasma)	Propionate and butyrate↓	/	204
Pan cancer	Cachectic vs non-cachectic patients/ HCs (human fecal)	Acetate↓	/	205
HCC	HCC mice vs WT mice (mice plasma)	Acetate↓(~50%)	Beneficial	62
NAFLD-HCC	NAFLD-HCC mice fed a HFHC diet vs control mice fed a HFLC diet (mice fecal)	Acetate/*Bifidobacterium pseudolongum*↓	Beneficial	38
NAFLD-HCC	NAFLD-HCC mice fed a HFHC diet vs control mice fed a HFLC diet (mice fecal)	Valerate/ *Lactobacillus acidophilus*↓	Beneficial	153
CRC	Responded vs nonresponded to ICB patients (human fecal)	*Roseburia intestinalis*↑	Beneficial	135
CRC	Responded vs nonresponded to neoadjuvant radiochemotherapy patients (human fecal)	SCFAs↑	Beneficial	206
NSCLC	Responded vs nonresponded to anti-PD-1 therapy patients (human plasma)	Butyrate↑	Beneficial	137
Pan cancer	Responded vs nonresponded to oxaliplatin patients (human plasma)	Butyrate↑	Beneficial	136
Pan cancer	Responded vs nonresponded to PD-1i therapy patients (human fecal)	SCFAs↑	Beneficial	162
Pan cancer	CR-treated mice vs IF-treated mice /mice fed ad libitum(mice fecal)	Acetate↑	Beneficial	61
CRC	CRC patients vs HCs (human fecal)	Formate/*Fusobacterium nucleatum*↑	Harmful	149
CRC	Responded vs nonresponded to capecitabine patients (human fecal)	Isobutyrate↓	Harmful	207
NSCLC	lung tumor tissues vs normal lung tissues	Acetate↑	Harmful	55
LC	Recurrence vs non-recurrence patients (human tumor tissue)	Butyrate↑	Harmful	104
LUAD	lung adenocarcinoma tissues vs normal lung tissues	Acetate↑	Harmful	69
Metastatic Melanoma	MM patients resistance to anti-CTLA-4 blocking mAbs (Human plasma)	Propionate and butyrate↑	Harmful	189

↑, upregulate. ↓, downregulate. Abbreviations: CRC, colorectal cancer; HCC, hepatocellular carcinoma; GC, gastric cancer; BC, Breast cancer; AML, acute myeloid leukemia; NSCLC, non-small cell lung cancer; NAFLD-HCC, non-alcoholic fatty liver disease-related hepatocellular carcinoma; LC, lung cancer; LUAD, lung adenocarcinoma; HCs, healthy controls; CRMM, complete remission multiple myeloma; RRMM, refractory disease multiple myeloma; IM, intestinal metaplasia; CSG, chronic superficial gastritis; WT, wild type; HFHC, high-fat/high-cholesterol; HFLC, high-fat/low-cholesterol; ICB, immune checkpoint blockade; CR, calorie restriction; IF, intermittent fasting; MM, Metastatic Melanoma; SCFAs, short-chain fatty acids.

## Abbreviations

SCFAs: short-chain fatty acids
TME: tumor microenvironment
HDAC: histone deacetylase
HAT: histone acetyltransferase
GPR41/43/109 A: G protein-coupled receptor 41/43/109 A
FMT: fecal microbiota transplantation
Abx: antibiotic mixture
ICIs: immune checkpoint inhibitors
ACT: adoptive T cell therapy
FAs: lipids, fatty acids
MCFAs: medium-chain fatty acids
LCFAs: long-chain fatty acids
MCFAs: medium-chain fatty acids
ROS: reactive oxygen species
CAFs: cancer-associated fibroblasts
ACLY: ATP citrate lyase
PDAC: pancreatic ductal adenocarcinoma
CODH: carbon monoxide dehydrogenase
ACSS: acetyl-CoA synthetase
THF: tetrahydrofolate
CRC: colorectal cancer
HCC: hepatocellular carcinoma
NAFLD-HCC: non-alcoholic fatty liver disease-related hepatocellular carcinoma
MCT1: monocarboxylate transporter 1
SMCT1: sodium-coupled monocarboxylate transporter 1
eEF1A1: eukaryotic elongation factor 1A1
GBM: glioblastoma
MAO-B: monoamine oxidase B
GSC: glioma stem cell-like cell
DLAT: dihydrolipoamide S-acetyltransferase
USP10: ubiquitin-specific peptidase 10
PD-L1: programmed death-ligand 1
IFN-γ: interferon gamma
ILC3s: type 3 innate lymphoid cells
HSC: hepatic stellate cell
Sox13: SRY-box transcription factor 13
ACC1: acetyl-CoA carboxylase 1
MDSCs: myeloid-derived suppressor cells
LUAD: lung adenocarcinoma
FFAR2: free fatty acid receptor 2
TCA: tricarboxylic acid
PD-1: programmed death-1
PEP: phosphoenolpyruvate
DHAP: dihydroxyacetone phosphate
TNFAIP1: tumor necrosis factor alpha-induced protein 1
HECTD2: HECT domain E3 ubiquitin protein ligase 2
EHMT2: euchromatic histone-lysine N-methyltransferase 2
EMT: epithelial-to-mesenchymal transition
MCEE: methylmalonyl-CoA epimerase
MMA: methylmalonic acid
ICD: immunogenic cell death
AML: acute myeloid leukemia
DAMPs: damage associated molecular patterns
DC: dendritic cell
MPN: myeloproliferative neoplasms
GLUT1: glucose transporter 1
G6PD: glucose-6-phosphate dehydrogenase
IGF-1: insulin-like growth factor-1
TCR: T-cell receptor
PBMCs: peripheral blood mononuclear cells
LC: lung cancer
SULF1: sulfatase 1
AhR: aryl hydrocarbon receptor
ECM: extracellular matrix
THF: tetrahydrofolate
SHMT: serine hydroxymethyltransferase
MTHFD: methylenetetrahydrofolate dehydrogenase
MLNs: mesenteric lymph nodes
CTLs: cytotoxic T lymphocytes
AN: Alaska Native
GC: gastric cancer
BC: breast cancer
NSCLC: non-small cell lung cancer
HCs: healthy controls
CRMM: complete remission multiple myeloma
RRMM: refractory disease multiple myeloma
IM: intestinal metaplasia
CSG: chronic superficial gastritis
WT: wild type
HFHC: high-fat/high-cholesterol
HFLC: high-fat/low-cholesterol
ICB: immune checkpoint blockade
CR: calorie restriction
IF: intermittent fasting
MM: Metastatic Melanoma
DOXO: doxorubicin
OXA: oxaliplatin
5-FU: 5-fluorouracil
CAR: chimeric antigen receptor
APL: acute promyelocytic leukemia
NED: neurodifferentiation
PCa: adenocarcinoma prostate cancer
ENZA: enzalutamide
PET: positron emission tomography
RA: retinoic acid
